# Visceral Endoderm Expression of Yin-Yang1 (YY1) Is Required for VEGFA Maintenance and Yolk Sac Development

**DOI:** 10.1371/journal.pone.0058828

**Published:** 2013-03-15

**Authors:** Siyeon Rhee, Mara-Isel Guerrero-Zayas, Mary C. Wallingford, Pablo Ortiz-Pineda, Jesse Mager, Kimberly D. Tremblay

**Affiliations:** Department of Veterinary and Animal Science, University of Massachusetts, Amherst, Massachusetts, United States of America; Sanford Burnham Medical Research Institute, United States of America

## Abstract

Mouse embryos lacking the polycomb group gene member Yin-Yang1 (YY1) die during the peri-implantation stage. To assess the post-gastrulation role of YY1, a conditional knock-out (cKO) strategy was used to delete YY1 from the visceral endoderm of the yolk sac and the definitive endoderm of the embryo. cKO embryos display profound yolk sac defects at 9.5 days *post coitum* (*dpc*), including disrupted angiogenesis in mesoderm derivatives and altered epithelial characteristics in the visceral endoderm. Significant changes in both cell death and proliferation were confined to the YY1-expressing yolk sac mesoderm indicating that loss of YY1 in the visceral endoderm causes defects in the adjacent yolk sac mesoderm. Production of Vascular Endothelial Growth Factor A (VEGFA) by the visceral endoderm is essential for normal growth and development of the yolk sac vasculature. Reduced levels of VEGFA are observed in the cKO yolk sac, suggesting a cause for the angiogenesis defects. *Ex vivo* culture with exogenous VEGF not only rescued angiogenesis and apoptosis in the cKO yolk sac mesoderm, but also restored the epithelial defects observed in the cKO visceral endoderm. Intriguingly, blocking the activity of the mesoderm-localized VEGF receptor, FLK1, recapitulates both the mesoderm and visceral endoderm defects observed in the cKO yolk sac. Taken together, these results demonstrate that YY1 is responsible for maintaining VEGF in the developing visceral endoderm and that a VEGF-responsive paracrine signal, originating in the yolk sac mesoderm, is required to promote normal visceral endoderm development.

## Introduction

Yin-Yang 1 (YY1) is aptly named because of its documented roles as a transcriptional activator and repressor, binding directly to DNA via a consensus-binding site or as part of repressive complexes. *In vitro* analysis has revealed that YY1 is required for appropriate regulation of a variety of basic cellular processes including proliferation, cytokinesis, epithelial-mesenchymal transition, apoptosis and DNA repair [1]. Based on these diverse roles in essential cellular processes in normal cells it is not surprising that inappropriate regulation of *Yy1* is believed to influence oncogenesis [2], [3]. Given the importance of YY1’s observed roles *in vitro* and its implication in a number of cancers, understanding the role of this gene in normal mammalian developmental processes is of great interest.

YY1 is the vertebrate homolog of the *Drosophila* pleiohomeotic (Pho), a member of the polycomb group (PcG) of proteins. Pho is an essential member of the multiprotein Polycomb Repressive Complex, providing DNA binding activity [4]. Mammalian YY1 can substitute for Pho in wing imaginal disc development and partially rescues Pho mutant fly embryos demonstrating that these essential PcG interaction and DNA binding functions are conserved in the mammalian protein [5]. Two high molecular weight PcG complexes, polycomb repressive complex 1 and 2 (PRC1 and 2), are conserved in vertebrates. Although YY1 has been shown to interact with vertebrate PRC2 complex members it remains unclear if YY1 targets PRC2 in mammalian cells [6].

YY1 is expressed ubiquitously in the extraembryonic and embryonic portions of the developing mouse embryo including the germ line and all adult tissues examined [7], [8], [9], [10]. Complete knockout of *Yy1* results in peri-implantation lethality demonstrating a critical early role for this gene [9]. The generation of a conditional allele has allowed for a better understanding of the tissue-specific requirements of YY1 in embryonic and adult lineages [11]. YY1 has been shown to play a critical role in immunity and B-cell lineage progression [12], [13], where knockout in B-cells produces arrest at the pro-B cell stage [14]. In the developing oocyte, loss of YY1 leads to a failure of oocyte-granulosa communication and a subsequent loss of fertility [7]. YY1 is also essential during gastrulation in the epiblast for appropriate primitive streak formation and proper regulation of the Nodal signaling pathway [8]. These recent studies have identified defects in paracrine signaling *in vivo* upon tissue-specific deletion of *Yy1*.

Here we show that YY1 expression in the visceral endoderm of the yolk sac regulates VEGF in this tissue. VEGF is essential for the growth and development of both the yolk sac and embryonic vasculature. *VegfA* heterozygous animals have embryonic and yolk sac angiogenesis defects that are apparent by 9.5 *dpc*, suggesting that VEGFA levels are critical for normal vasculature development [15], [16]. Furthermore, reductions in VEGFA during early postnatal development affect embryo and organ size, revealing that the appropriate levels VEGFA signaling is also essential throughout postnatal development [17]. Finally, the reduction of *VegfA* in the visceral endoderm alone results in yolk sac angiogenesis defects, suggesting that the level of VEGFA produced by the visceral endoderm is responsible for angiogenesis in the underlying mesoderm [15], [18].

While most of the *VegfA* in the yolk sac is produced by the visceral endoderm, both of the cognate receptors, including *Flt1* (*VegfR1*) and *Flk1* (*VegfR2/Kdr*) are expressed specifically in the adjacent yolk sac mesoderm [19], [20], [21]. Consistent with these findings, knock-out analysis of *Flt1* and *Flk1* demonstrate an essential role for these receptors in blood vessel development [22], [23], [24].

Here we use the transgenic *FoxA3-Cre* line [25] to conditionally delete *Yy1* in the visceral endoderm of the yolk sac and in the embryonic definitive endoderm. While the initiation of early endoderm organogenesis is only slightly delayed, yolk sac development is severely disrupted. At 9.5 *dpc* the mutant yolk sac is morphologically abnormal and angiogenesis, which occurs in the adjacent *Yy1*-expressing yolk sac mesoderm derivatives, is disrupted. A variety of visceral endoderm defects are observed at 9.0 *dpc*, including a loss of large apical lysosomes as well as changes in visceral endoderm-specific gene expression. Although the level of *VegfA* transcripts remain unchanged in the cKO yolk sac, VEGFA protein is dramatically reduced by 9.25 *dpc*, suggesting that YY1 regulates VEGFA translation or stabilization. Surprisingly, we show that exogenous VEGF rescues the *Yy1* cKO defects in both yolk sac layers and propose that a paracrine signal produced by the VEGF-receiving YS mesoderm is required to maintain key visceral endoderm characteristics. Taken together these data support a critical role for YY1 in maintenance of VEGF levels and highlight a new role for VEGF-responsive yolk sac mesoderm-derived tissue in supporting visceral endoderm function.

## Materials and Methods

### Ethics Statement

All animal studies were approved by the Institutional Animal Use and Care Committee, University of Massachusetts, Amherst protocol #2012-043.

### Mouse Breeding Scheme and Genotyping

Females homozygous for the *Yy1* floxed allele (*Yy1^fl/fl^*) [11] were mated to males heterozygous for a *Yy1* null allele (*Yy1^+/Δ^*) and for the *Foxa3-Cre* transgene (*FoxA3-Cre)* [25] to obtain *Yy1^fl/Δ^; FoxA3-Cre* embryos. The morning of the copulation plug was defined as 0.5 *dpc*. After obtaining embryos at the desired stage, a portion of the yolk sac or embryo used for PCR genotyping using the primers of *Yy1*∶5′ACCTGGTCTATCGAAAGGAAGCAC3′, 5′GCTTCGGCTATTCCTCGCTCATAA 3′ and 5′CCAAAGTTCGAAACCTGCTTTCCT3′; *Cre*: 5′CATTTGGGCCAGCTAAACAT3′ and 5′ATTCTCCCACCGTTACG3′. *Yy1^fl/Δ^; FoxA3-Cre* embryos are referred to as “mutants” or “cKO” and other genotypes referred to as “wild-type” (WT) for simplicity.

### Histology, Immunofluorescence and Immunohistochemistry

Embryos and yolk sacs were dissected and fixed in 4% paraformaldehyde (PFA)/PBS overnight at 4°C. The following day they were washed in PBS, dehydrated in an ascending methanol sequence, xylene treated, embedded in paraffin and sectioned at 7.5 µm. For histological analysis, routine hematoxylin and eosin (H&E) staining was performed on dewaxed slides.

For immunofluorescence (IF) slides were dewaxed in xylenes, rehydrated with EtOH, and subjected to antigen retrieval in Tris buffer pH 10.0 for 10 min. The slides were then washed in PBT and incubated in blocking buffer (0.5% milk powder, 99.5% PBT) for 2 hrs at room temperature and then with primary antibody in blocking buffer at 4°C overnight in a humid chamber. Slides were then washed three times with PBT, incubated for 1 hr with secondary antibody in blocking buffer at room temperature. Nuclei were countered stained with 4′, 6-diamidino-2-phenylindole dihydrochloride (DAPI, Molecular Probes, 1∶10,000) for 3 min and then coverslipped with Prolong Gold Antifade Reagent (Invitrogen). Sections were imaged on a Nikon Eclipse TE2000-S inverted microscope with Retiga EXi Fast camera with NIS Elements imaging software. Primary antibodies used include: rabbit anti-YY1 [1∶100, Santa Cruz (sc-1703)]; goat anti-HNF4α [1∶100, Santa Cruz (sc6556)]; mouse anti-CDH1 [1∶500, BD Bioscience (610811)], mouse anti-αSMA [1∶500, Sigma (A2547)], rabbit anti-cleaved Caspase-3 [1∶500, Abcam (ab13847)], rabbit anti-phosphohistone-H3 [1∶500, Abcam (Ab5176)], rabbit anti-VEGFA [1∶100, Santa Cruz (A120)] and guinea pig anti-PDX1 [1∶1000, Abcam (ab47308)]. Secondary antibodies (Molecular Probes) were used at 1∶500. IF of IgG was performed as detailed above except that a primary antibody was omitted and the secondary antibody was Donkey anti-mouse [1∶500, Molecular Probe (A11036)].

Whole-mount IF of PECAM [1∶50, Pharmingen (553369)] and αSMA (1∶100) were performed as described [26]. Following IF, yolk sacs were imaged and coverslipped on glass slides using a Nikon SMZ1500 microscope equipped with a MicroPublisher 5.0 RTV camera and Q-imaging software.

For immunohistochemistry (IHC), deparaffinized slides were subjected to 1% H_2_O_2_ for 30 min., rinsed in PBS and then blocked in PBS-0.1% Triton X-100 for 30 min. Slides were then incubated overnight at 4°C in blocking buffer as above containing primary antibodies including rabbit anti-YY1 [1∶200, Santa Cruz (sc-1703)], goat anti-HNF4α [1∶200, Santa Cruz (sc6556)]. After three washes in PBS, slides were incubated for 1 hr in blocking buffer containing the appropriate biotinylated secondary antibody (Vector Labs) at 1∶500. The secondary was detected with Vectastain Elite ABC kit (Vector Labs) and stained using DAB (Vector Labs). For IgG localization, biotinylated anti-mouse IgG (Vector Labs) was used as outlined above except that the primary antibody was omitted. After ICH, slides were coverslipped with mounting media [Richard-Allan Scientific (8310-16)] and visualized using a Nikon Eclipse TE2000-S inverted microscope with Retiga EXi Fast camera with color filters and NIS Elements imaging software.

For *LacZ* staining, embryos double heterozygous for the *R26R* allele [27] and *FoxA3Cr*e allele were stained and processed as reported [28].

### Western Blot Analysis

5–7 yolk sacs for each sample were pooled and lysed by extraction buffer with Complete Protease Inhibitor Cocktail (Roche) and Phosphatase Inhibitor Cocktail (Roche). The samples were homogenized and soluble proteins obtained by centrifugation. Equal amounts of protein were loaded onto a 4%–20% gradient Tris-Glycine gel under reducing conditions. After transferring onto 0.45 µm PVDF membranes (Millipore) following standard protocols, the membranes were blocked (5% nonfat dry milk in TBS with 0.1% Tween20) for 1 hr and incubated overnight at 4°C with rabbit anti-VEGFA [1∶1000, Santa Cruz (A120)], followed by secondary (1∶5000, Jackson Immuno Research) for 1 hr. Results were visualized using ECL reagent (Amersham Life Sciences). Blots were stripped and reprobed with mouse anti-GAPDH [1∶5000, Millipore (MAB374)] as a loading control. Autoradiographs were quantified using Image J software (NIH).

### RNA Extraction, cDNA Synthesis and PCR

Whole yolk sacs or yolk sac layers (used only in Fig. S3) were dissected and saved in RNA Later (QIAGEN) overnight at 4°C then stored at 80°C. The yolk sac layers were separated using the trypsin/pancreatin method as described [29]. Total RNA was extracted using the High Pure RNA Isolation kit (Roche) according to manufacturer’s recommendation. 500 ng of RNA was reverse transcribed using the iScript ™ cDNA Synthesis Kit (Bio-Rad) according to the manufacturers’ instructions. RT-PCR was performed with 36 cycles of 30 sec at 60°C, 72°C and 94°C. *Hprt* and *β-actin* were used as internal controls. All primer sets for traditional RT-PCR used are listed (Table S1). Quantitative RT-PCR (qPCR) was performed using the following Taqman gene expression Assays: *Yy1* (MM0456392_m1), *VegfA* (MM00437304_m1) and *Hnf4α* (MM00455964_m1). These assays were multiplexed with *ActB* using PerfeCTa® qPCR SuperMix, Low ROX™ (Quanta Biosciences). Reactions were performed on a Stratagene 3001 mx qPCR machine using Quanta’s recommend cycling conditions.

### Apoptosis and Proliferation

3 WT and 3 cKO yolk sacs at 8.5 and 9.0 *dpc* were subjected to IF (as noted above) using either cleaved Caspase-3/CDH1/DAPI to assess apoptosis or PH3/CDH1/DAPI to assess proliferation. CDH1 was used to identify the visceral endoderm layer and DAPI used to distinguish individual cells. A total of 2–3000 cells were counted from 10–12 independent fields for each data point. Image-J was used to assist with counting. All statistical analysis performed on these samples and elsewhere were performed by comparing averages under the null hypothesis that there are no differences between WT and cKO samples with the student’s T-test. Statistical differences were measured by two-tailed Student’s *t* test analysis of variance.

### Blood Vessel Measurements

Blood vessel size was determined by counting the same number of blood vessels on H & E stained sections from 3 mutant and 3 WT yolk sacs using the NIS Elements line measurement tool. Blood vessel density was assessed by obtaining the average of all vessels counted from the same total area from 3 WT and 3 mutant embryos.

### LysoTracker Staining

Whole embryos with intact yolk sacs were dissected and incubated with 100 nM LysoTracker Red (Invitrogen) in PBS at 37°C for 5 min. Yolk sacs were mounted on a glass bottom culture dishes and immediately imaged.

### Electron Microscopy

Yolk sacs (n = 2 for each age/genotype combination) were fixed with 2.5% glutaraldehyde in PBS at 4°C overnight. Samples were post-fixed in 2% osmium tetroxide in 0.1 M PBS pH 7.4, dehydrated in a graded series of acetone (10% steps) and embedded in epoxy resin. The resin was polymerized at 70°C for 12 hr. Ultrathin sections of 60 nm were cut with a Reichert Ultracut E Ultramicrotome and placed on copper grids. Sections were then counterstained with uranyl acetate and imaged with a JEOL 100 S electron microscope.

### Whole Embryo Culture

Litters were dissected at 8.5 *dpc* and cultured until 9.5 *dpc* (26–28 hrs) as previously described [30]. VEGF [Peprotech (100-20C)] or SU1498 (Millipore) was diluted in DMSO and added directly to the culture media to obtain 200 ng/ml (VEGF) or 40 µM (SU1498), while an equal amount of DMSO-alone was added to the controls. After culture all embryos were photographed and processed for histology. Because the cKO embryos are not apparent at the onset of culture, litters were blindly divided into VEGF treatment groups and the ectoplacental cones dissected at the end of culture for genotyping.

## Results

### *Yy1* is Required for Yolk Sac Development

**Figure 1:**
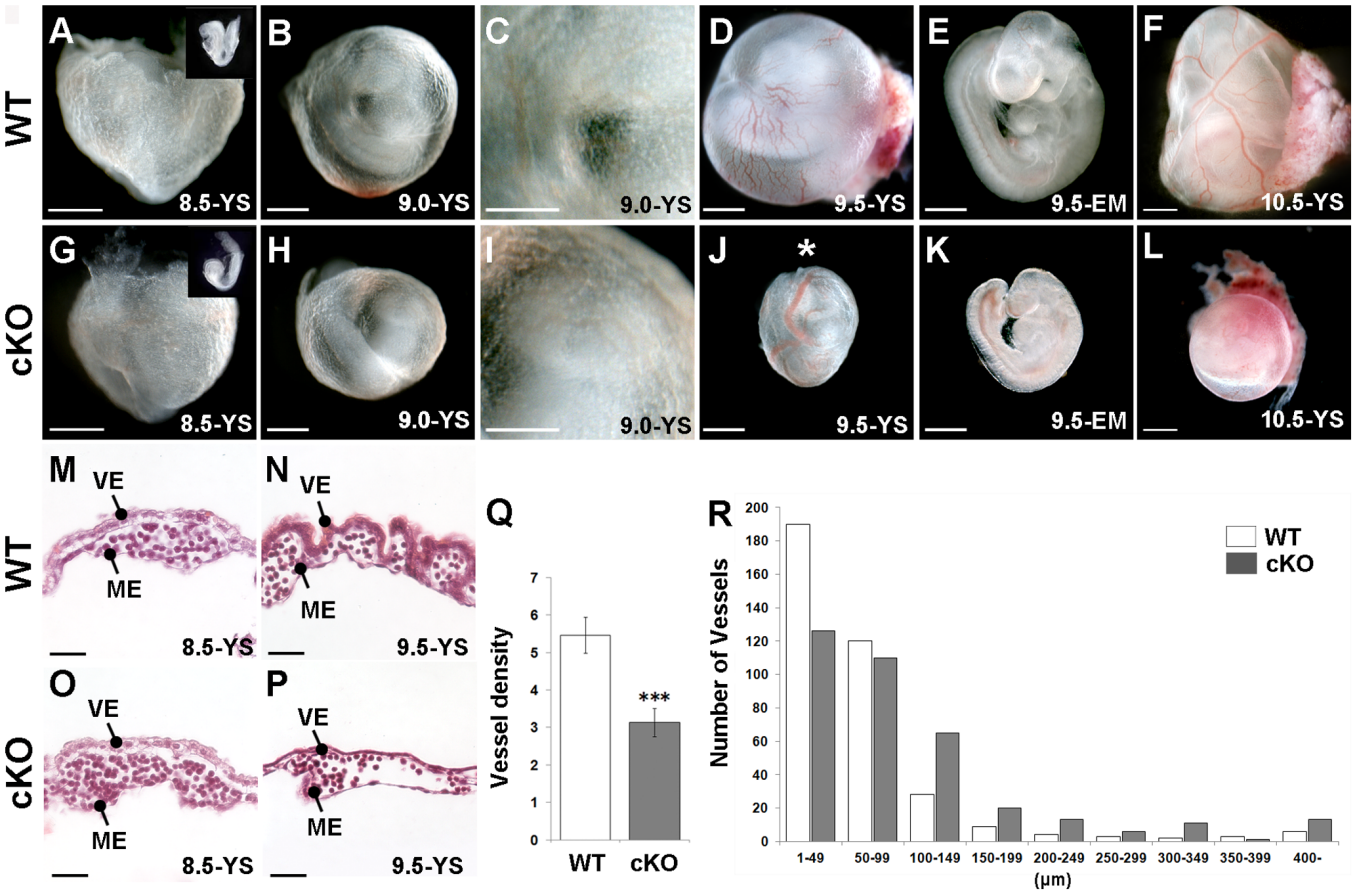
*FoxA3-Cre* mediated *Yy1* cKO deletion results in prominent yolk sac defects at 9.5 *dpc*. A-L) Bright field images of WT (A–F) and cKO (G–L) embryos (EM) alone or embryos within their yolk sacs (YS) at the indicated stages. A, G) 8.5 *dpc* mutant embryos (inset in G) are sometimes slightly delayed compared with WT (inset in A) but display no noticeable yolk sac defects. B–C, H–I) 9.0 *dpc* mutants display relatively normal yolk sac blood vessel development. D–E, J–K) 9.5 *dpc* mutant yolk sacs have dilated vessels (asterisk in J) and poor vessel organization (compare D to J). cKO embryos (K) are smaller than WT embryos (E) from the same litter. F, L) While prominent large blood vessels are easily detected in 10.5 *dpc* WT yolk sacs (F), the yolk sacs of mutants are uniformly pale (L). M–P) A comparison of WT and mutant H&E stained yolk sac sections demonstrates that while no differences are found at 8.5 *dpc* (M, O), the 9.5 *dpc* cKO yolk sac (P) has fewer and larger vessels compared with WT (N). Q) Investigation of the same sized area at 9.5 *dpc* revealed significantly fewer vessels in mutant compared with WT yolk sacs (*** = p<0.001; error bar = standard error). R) A size distribution chart at 9.5 *dpc* reveals that mutants contain fewer of the small vessels (<100 µm) and more of the larger vessels (>100 µm) compared with WT yolk sacs.

To examine the role of *Yy1* in the visceral and definitive endoderm, we used a conditional deletion strategy employing a *FoxA3-Cre* transgene and a conditional *Yy1* allele [11], [25]. *FoxA3-Cre* mediated deletion of *Yy1* (referred to as “mutant”, “cKO” or “*Yy1* cKO”) resulted in an embryonic delay occasionally observed by 8.5 *dpc* (compare inset in Fig. 1A to inset in G), frequently observed by 9.0 *dpc* and always found in cKO embryos by 9.5 *dpc* (compare Fig. 1E to K). Between 8.5–9.0 *dpc* the cKO yolk sac is morphologically similar to WT (compare Fig. 1A to G, B–C to H-I and M to O). Compared with WT, the 9.5 *dpc* cKO yolk sac displayed abnormal vasculature including dilated vessels and a paucity of vasculature (compare Fig. 1D and N to J and P). The overall numbers of vessels per area of yolk sac was significantly lower in the mutant (Fig. 1Q) and there were fewer vessels smaller than 100 µm and more vessels larger than 100 µm when compared with a WT size distribution (Fig. 1R). By 10.5 *dpc*, cKO embryos were considerably smaller than WT and displayed a pale thin yolk sac (Fig. 1F, L). Finally, because blood vessel development is dependent upon appropriate hemodynamic flow [31], [32], it is important to note that cKO embryos do contain a beating heart through 10.5 *dpc* (data not shown). At 9.0 *dpc*, blood flow in the cKO yolk sac is indistinguishable from that of WT, although flow is impeded in the cKO yolk sacs at 9.5 *dpc*, coincident with the observed vascular defects (data not shown).

**Figure 2:**
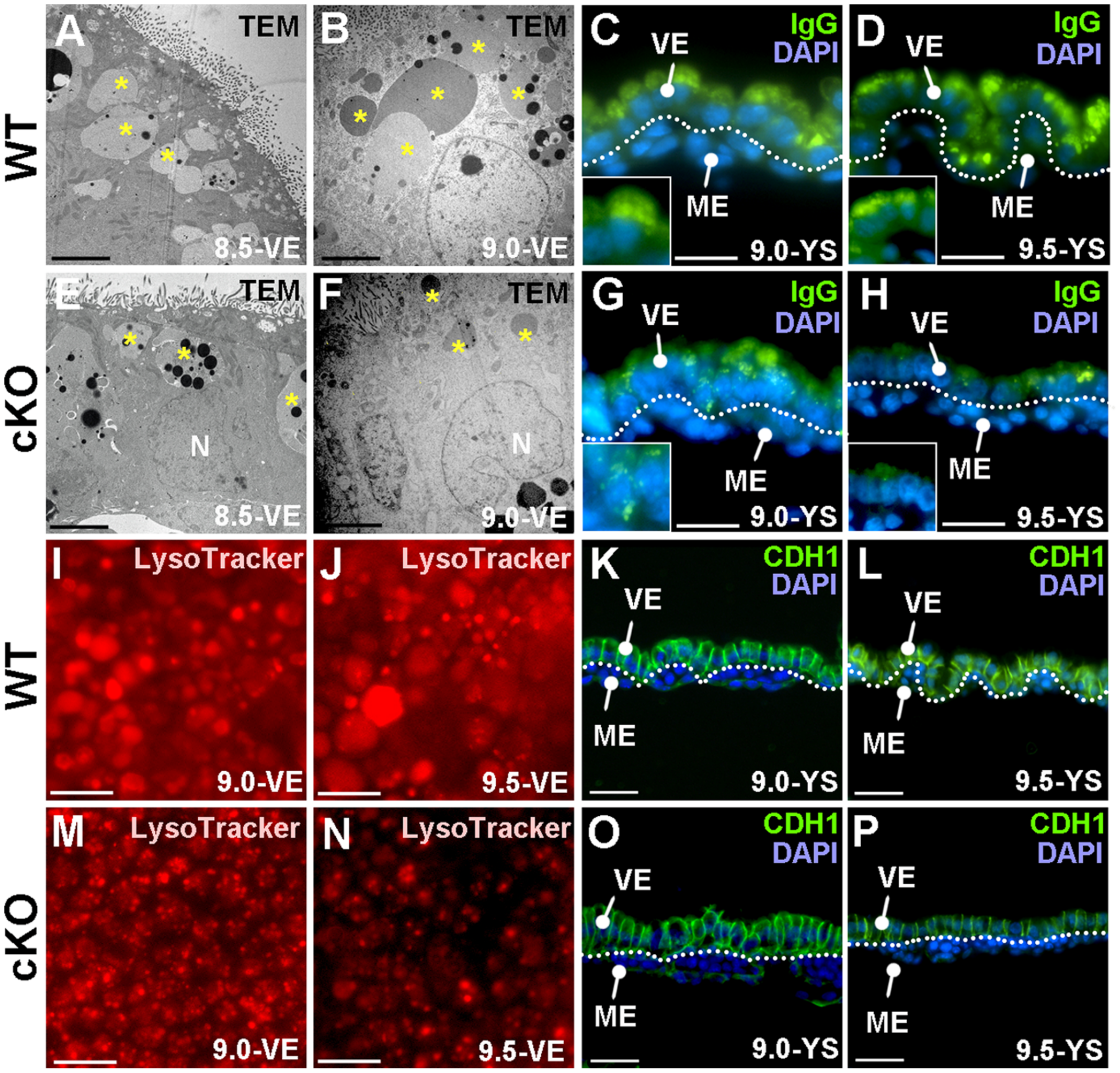
*Yy1* cKO visceral endoderm displays a reduction of apical lysosome size and other epithelial characteristics. A–B, E–F) Transmission electron microscopy (TEM) of WT (A–B) and cKO (E–F) visceral endoderm (VE) sections reveals large apical lysosomes in 8.5 and 9.0 *dpc* WT visceral endoderm (asterisks, A–B) and in 8.5 *dpc* cKO visceral endoderm (asterisks, E). At 9.0 *dpc* the size of the apical lysosomes are greatly reduced in the cKO (compare asterisks, in F to B). C–D, G–H) IgG localization (green) at the apical surface is readily noted in 9.0–9.5 *dpc* WT yolk sac sections (YS, C–D) while IgG distribution is reduced in the mutant at the same stages (G–H). Inset is a higher magnification view of a portion of the visceral endoderm. I–J, M–N). Whole-mount LysoTracker Red staining reveals large filled lysosomes from 9.0–9.5 *dpc* (I–J) in WT tissue while the LysoTracker-filled areas are reduced in the mutant samples (M–N). K–L, O–P) Immunolocalization of E-Cadherin (CDH1; green) reveals epithelial cell-cell adhesions in the visceral endoderm of WT yolk sac sections from 9.0–9.5 *dpc* (K–L). CDH1 expression is slightly reduced at 9.0 *dpc* and more profoundly reduced at 9.5 *dpc* in cKO visceral endoderm (O–P). ME = mesoderm; N = nucleus.

To gain insight into how *Yy1* loss affects the visceral endoderm, we examined the ultrastructure of this tissue using electron microscopy. While the 8.5 *dpc* cKO visceral endoderm appears morphologically normal when compared with WT (Fig. 2A, E), by 9.0 *dpc* the large apical lysosomes observed in WT are dramatically reduced in size in cKO tissue (asterisks in Fig. 2B, F). IgG localization, which accumulates in the apical regions of the visceral endoderm, and LysoTracker Red, a fluorescent cell-permeable probe that accumulates in the lysosomes of live yolk sacs, were used to further examine the alterations in lysosome size. In agreement with the TEM results, 9.0 *dpc* cKO embryos had reduced levels of IgG and a noticeable reduction in the size of LysoTracker filled vesicles compared with WT at 9.0 *dpc* (compare Fig. 2C to G and I to M) and both are more noticeably reduced by 9.5 *dpc* (compare Fig. 2D to H and J to N). Since the apically located vesicles are a specialized characteristic of absorptive epithelium, we also examined more general epithelial characteristics such as the cell-cell adhesion marker E-Cadherin (CDH1) and found that it too was slightly downregulated at 9.0 *dpc* compared with WT (Fig. 2K, O) and more profoundly downregulated by 9.5 *dpc* (Fig. 2 L, P). Taken together these data indicate that the epithelial characteristics of the cKO visceral endoderm are disrupted by 9.0 *dpc*, before gross morphological defects are evident.

### Loss of YY1 Occurs First in Visceral Endoderm and then in Definitive Endoderm

**Figure 3:**
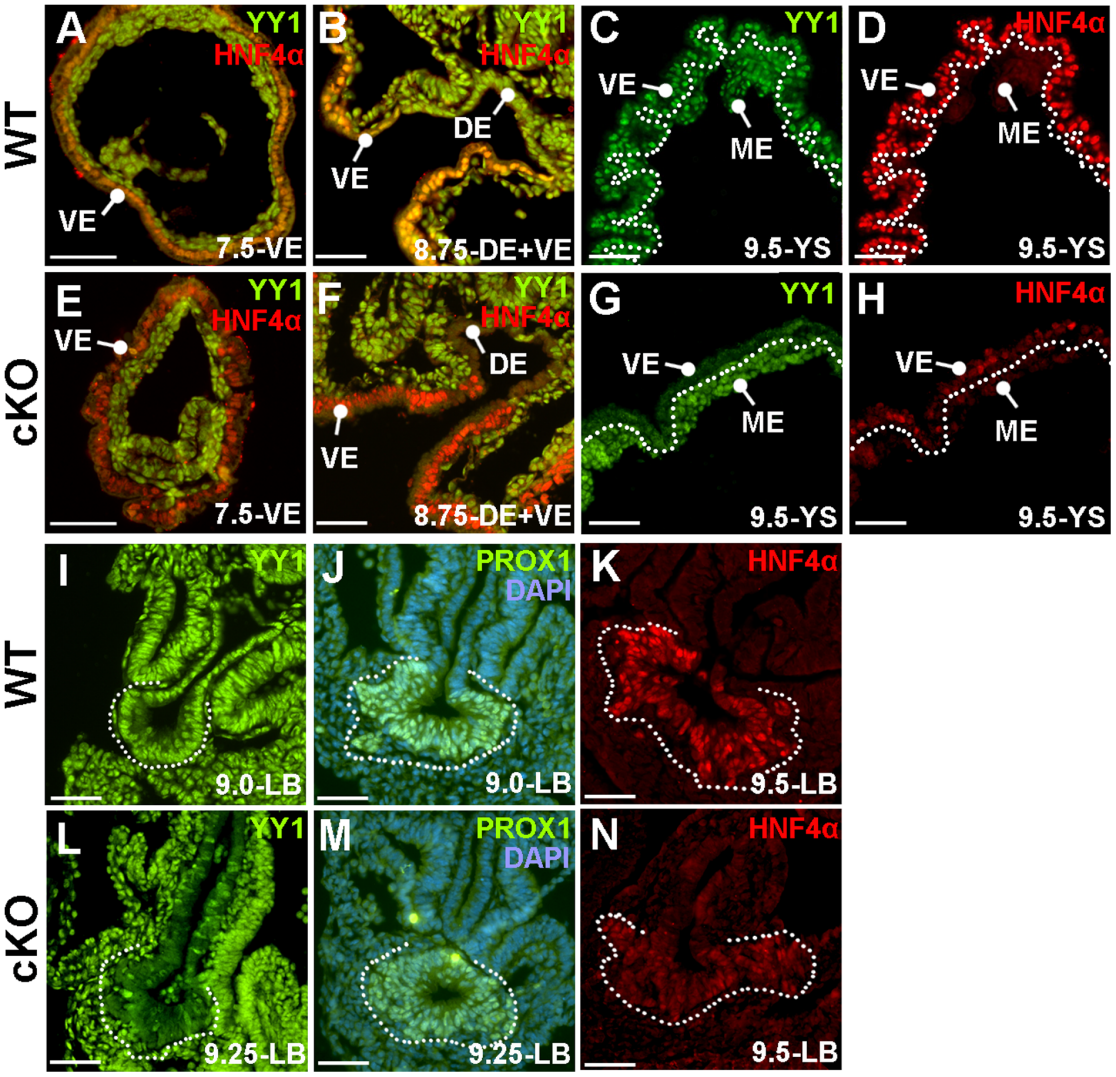
Efficient excision of YY1 in definitive and visceral endoderm is accompanied by reduced HNF4α. Immunofluorescence analysis of sectioned WT (A–D, I–K) and cKO tissue (E–H, L–N) at the stages indicated. A–C, I) YY1 (green) is ubiquitous in WT embryonic and extraembryonic tissues. A–B, D, K) HNF4α (red, orange when co-expressed with YY1) labels the visceral endoderm (A–B, D) and the developing liver bud (K). E–G, L) In cKO embryos, YY1 is downregulated in the extraembryonic visceral endoderm (VE) at 7.5 *dpc* (E) and is completely lost in the embryonic visceral endoderm by 8.75 *dpc* (F), when YY1 is also depleted in the definitive endoderm (DE) of the foregut. By 9.25 *dpc* YY1 is lost in most cells of the liver bud (L). E–F, H, N) Although HNF4α is present in the YY1-deficient visceral endoderm until 8.75 *dpc* (E, F) it is greatly reduced in both the visceral endoderm and in the nascent liver bud by 9.5 *dpc*. J, M) Despite the loss of YY1 in the nascent liver bud, the liver bud differentiation marker PROX1 is maintained in the cKO liver bud (M) at levels comparable to that observed in WT (J). The dotted line in C–D and G–H represent the division between the visceral endoderm and mesoderm derivative of the yolk sac, while in I–N the dashed line outlines the liver bud (LB).

YY1 is ubiquitously expressed in embryonic and in extraembryonic tissues during all stages examined (7.5–9.5 *dpc*, Fig. 3 A–C, I). To gain a better understanding of the progression of the cKO phenotype, we examined YY1 expression in cKO tissues. We find that at 7.5 *dpc* YY1 in the Hepatocyte Nuclear Factor 4α (HNF4α) expressing visceral endoderm is downregulated (Fig. 3E), and is entirely depleted in the visceral endoderm by 8.75 *dpc* (Fig. 3 F–G). We confirmed the visceral endoderm expression of the *FoxA3-Cre* transgene using the R26R allele [27] and found that the *Cre*-driven *LacZ* expression mimicked the loss of YY1 (Fig. S1).

Loss of YY1 in the definitive endoderm is initiated by 8.5 *dpc* and is widespread between 8.5 *dpc*-9.5 *dpc* (Fig. 3F, L and Fig. S1B–C, E–F). To determine if differentiation of the definitive endoderm was impaired in cKO embryos we examined liver and pancreas development. Although *Yy1* cKO embryos were often delayed, when the cKO embryos were compared to somite-matched embryos, *Yy1* cKO endoderm displayed appropriate early markers such as PROX1 in the liver bud (compare Fig. 3J to M) and PDX1 in the ventral and dorsal pancreas buds (Fig. S2). As observed in the visceral endoderm (Fig. 3H), HNF4α expression is downregulated at 9.5 *dpc* in the cKO liver bud (compare Fig. 3K to N).

### Angiogenesis Defects in Yolk Sac Mesoderm and Loss of VEGF

**Figure 4:**
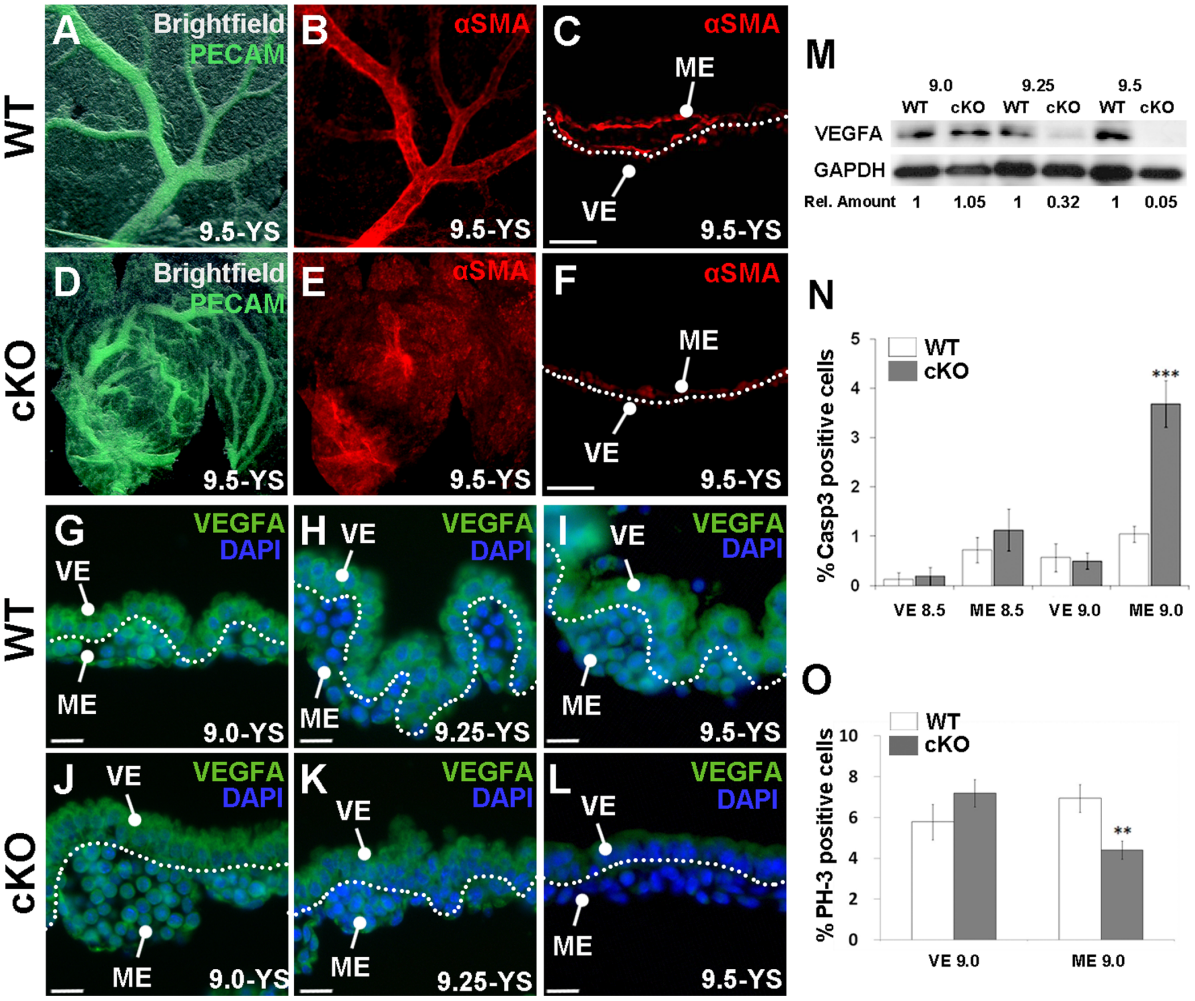
cKO embryos display a variety of defects in the yolk sac mesoderm. A–B, D–E) Whole mount immunofluorescence of 9.5 *dpc* WT (A–B) or mutant (D–E) yolk sacs (YS) using the endothelial marker PECAM (green) and the vascular smooth muscle marker (αSMA) demonstrates that the large disorganized vessels in the cKO (D) are not surrounded by αSMA (E). C, F) Section immunofluorescence of WT (C) and cKO (F) 9.5 *dpc* yolk sacs demonstrates loss of αSMA in the cKO. G–L) Section immunofluorescence of VEGFA (green) demonstrates relatively uniform VEGF levels in the 9.0, 9.25 and 9.5 *dpc* WT yolk sac (G–I) while VEGF distribution in the visceral endoderm of the mutant is progressively diminished at each stage (J–L). M) A Western blot of whole yolk sacs at the indicated stages. The ratio of VEGFA to GAPDH signal intensities for the cKO relative to each stage-matched WT control is displayed under each band. N) Cleaved Caspase-3 staining was used to assess the percentage of cell death in the yolk sacs layers of WT and cKO sections at 8.5 and 9.0 *dpc*. A significant increase in apoptosis was observed in the cKO mesoderm (ME) at 9.0 *dpc*. O) Phosphohistone-H3 (PH-3) staining was similarly used to assess proliferation and a significant decrease in proliferation was found in the cKO yolk sac mesoderm at 9.0 *dpc*. *** = p<0.001, ** = p<0.01; error bars = standard error; dotted line is drawn between the visceral endoderm (VE) and mesoderm derivatives (ME) on yolk sac sections.

It is well documented that inductive signals between the visceral endoderm and mesoderm derivatives of the yolk sac are required to coordinate development and growth of this vital extraembryonic tissue [18], [33], [34], [35]. To further investigate the defects observed in the yolk sac mesoderm, we examined expression of two markers associated with vascular development. The endothelial cell marker, PECAM, delineates the large primary vessels as well as the smaller secondary vessels in the yolk sac of WT 9.5 *dpc* (Fig. 4A). PECAM expression in cKO yolk sacs demonstrates that although endothelial cells have formed vessels, they are not well organized (Fig. 4D). α-Smooth muscle actin (αSMA), which is expressed in the smooth muscle that surrounds mature vessels, is highly expressed in large mature vessels in WT yolk sac (Fig. 4B–C). In the 9.5 *dpc* cKO yolk sac, we observe no αSMA, suggesting a defect in the later stages of blood vessel remodeling (Fig. 4E–F).

Based on its essential role in vascular development, we next examined VEGFA protein levels and distribution in cKO and WT yolk sacs. Immunofluorescence reveals that VEGFA is localized to the visceral endoderm and, at lower levels, in the underlying mesodermal tissue from 9.0–9.5 *dpc* (Fig. 4G–I). In cKO yolk sacs, VEGFA levels in the visceral endoderm appear slightly reduced at 9.0 *dpc*, more discernibly reduced at 9.25 *dpc* and is dramatically reduced by 9.5 *dpc* (Fig. 4J–L). Western blot analysis of pooled wild-type and mutant yolks reveals that absolute VEGFA levels are unchanged between samples at 9.0 *dpc*, reduced to one-third wild-type levels in mutants by 9.25 *dpc* and almost completely lost in the mutant by 9.5 *dpc* (Fig. 4M).

### Analysis of Cell Death and Proliferation in the cKO Yolk Sac

Because YY1 has been implicated in cell cycle regulation [11], we next examined the role YY1 has on YS proliferation and cell death. Phosphohistone-H3 (PH3) and cleaved Caspase-3 were used as markers of proliferation and apoptosis, respectively. Analysis of sectioned WT and mutant yolk sacs revealed no differences in proliferation (data not shown) or apoptosis at 8.5 *dpc* (Fig. 4N). At 9.0 *dpc*, loss of YY1 in the visceral endoderm resulted in an increased percentage of cleaved Caspase-3 positive cells and a decrease in the percentage of proliferating cells in the YY1-positive yolk sac mesoderm (Fig. 4N–O). Combined these data demonstrate that cKO of *Yy1* in the visceral endoderm results in proliferation and apoptosis defects in the adjacent yolk sac mesoderm.

### Examination of cKO Yolk Sac Gene-Expression

**Figure 5:**
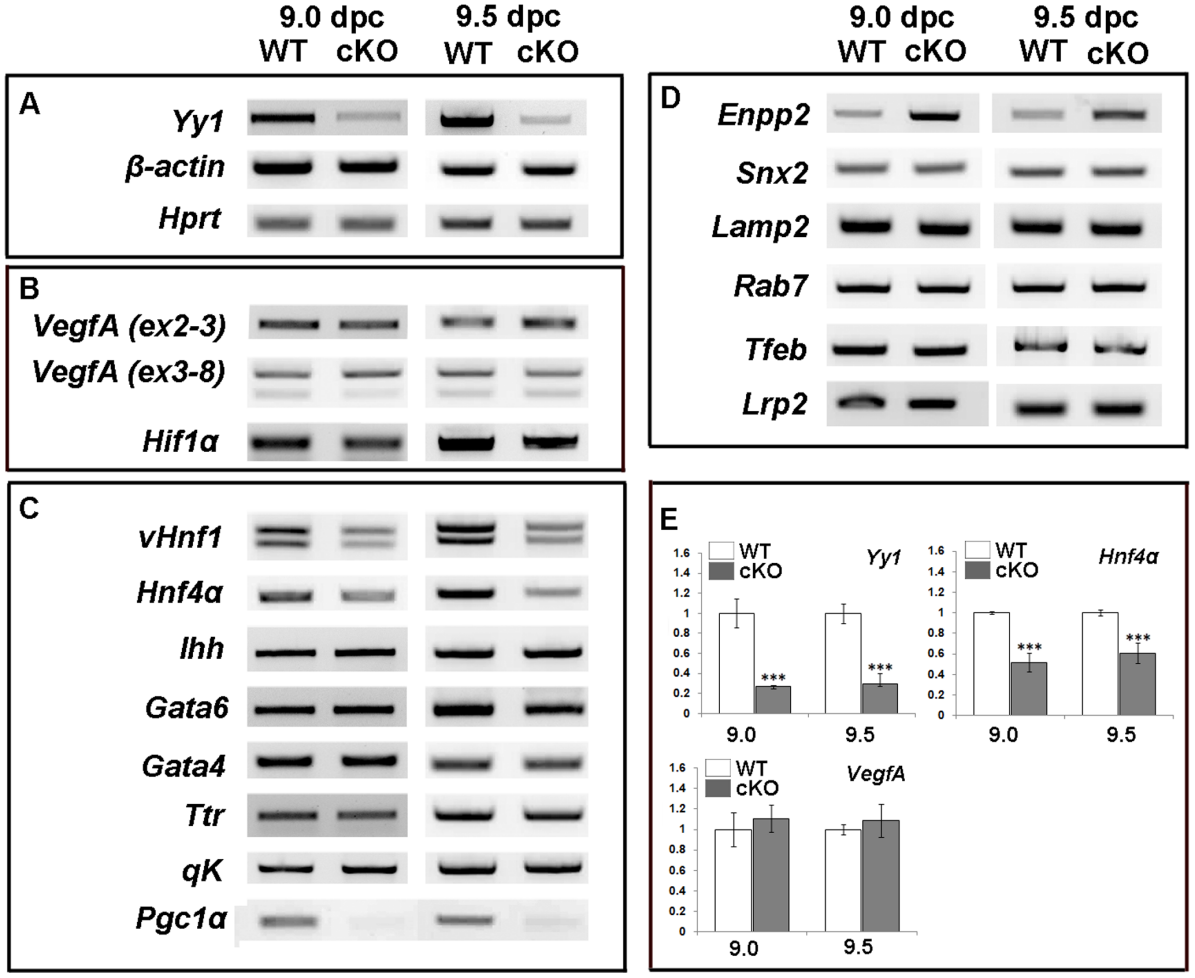
Changes in yolk sac gene expression in cKO embryos. A–E) RT-PCR and qPCR performed with cDNA prepared from whole 9.0 and 9.5 *dpc* cKO and WT yolk sacs. A) As expected, *Yy1* is significantly downregulated in whole cKO yolk sacs. *β-actin* and *Hprt* expression are used as loading controls. B) No expression differences between WT and cKO samples are noted for *VegfA* using primers that recognize all (Exon 2–3) or the alternative *VegfA* isoforms (Exons 3–8) nor in the *Vegf* transcriptional regulator *Hif1α*. C) While many visceral endoderm-specific genes show no expression differences, expression of *vHnf1*, *Hnf4α* and *Pgc1α* were all downregulated in cKO samples when compared to WT at 9.0 and 9.5 *dpc*. D) An examination of genes involved in lysosome biogenesis reveals no expression differences between WT and cKO yolk sacs with the exception of *Enpp-2*, which is upregulated in mutant samples at both stages examined. E) qPCR reveals that *Yy1* is expressed at ∼30% of WT levels in whole yolk sacs, where mesoderm derivatives maintain *Yy1*. qPCR was used to confirm that *VegfA* expression is not significantly altered between cKO and WT and that expression of the visceral endoderm gene, *Hnf4α* is significantly downregulated in cKO yolk sacs. *** = p value<0.001; error bars = standard error.

Appropriate VEGF signaling is critical for many aspects of vascular development in both the embryo and extraembryonic tissues including vascular remodeling and for eliciting an anti-apoptotic response [15], [16], [22], [23], [24]. At 9.5 *dpc VegfA* is mainly expressed in the visceral endoderm while the cognate VEGFA receptors, *Flk-1* and *Flt-1*, are expressed exclusively in the underlying mesoderm (Fig. S3 and [19], [20], [21]). RT-PCR and qPCR were used to examine *VegfA* expression in whole yolk sacs at 9.0–9.5 *dpc*. Surprisingly, we observed no change in *VegfA* mRNA at either 9.0 or 9.5 *dpc* (Fig. 5B, E), despite the reduction in protein. *Hif1α*, a transcriptional activator of the *VegfA* locus [36] is also maintained at normal levels in the cKO yolk sac (Fig. 5B).

Other essential VE-specific markers such as *Hnf4α*, *Ihh, vHnf1, Gata4, Gata6, Ttr* and *qK* were examined (Fig. 5C) and only *vHnf1* and *Hnf4α*, two transcription factors required for normal visceral endoderm differentiation [37], [38], [39], were altered in the cKO between 9.0–9.5 *dpc* (Fig. 5C, E). *Pgc1α* was recently shown to be downregulated in YY1-deficient muscle [40]. We found that *Pgc1α*, which is expressed exclusively in the visceral endoderm of the yolk sac (Fig. S3), is reduced in the cKO yolk sac at 9.0 and 9.5 *dpc* (Fig. 5C).

Based on the lysosome defects observed in mutant yolk sacs, we examined the expression of a several genes involved in lysosome biogenesis. No changes were found in *Snx2*, *Lamp2*, *Rab7*, *Tfeb* or *Lrp2* (Fig. 5D). However *Enpp-*2, which encodes for the exoenzyme Autotaxin and is required in the visceral endoderm for appropriate apical lysosome formation [41], is upregulated in cKO tissue at both 9.0 and 9.5 *dpc* compared with WT controls (Fig. 5D).

### Exogenous VEGF Rescues cKO Yolk Sac

**Figure 6:**
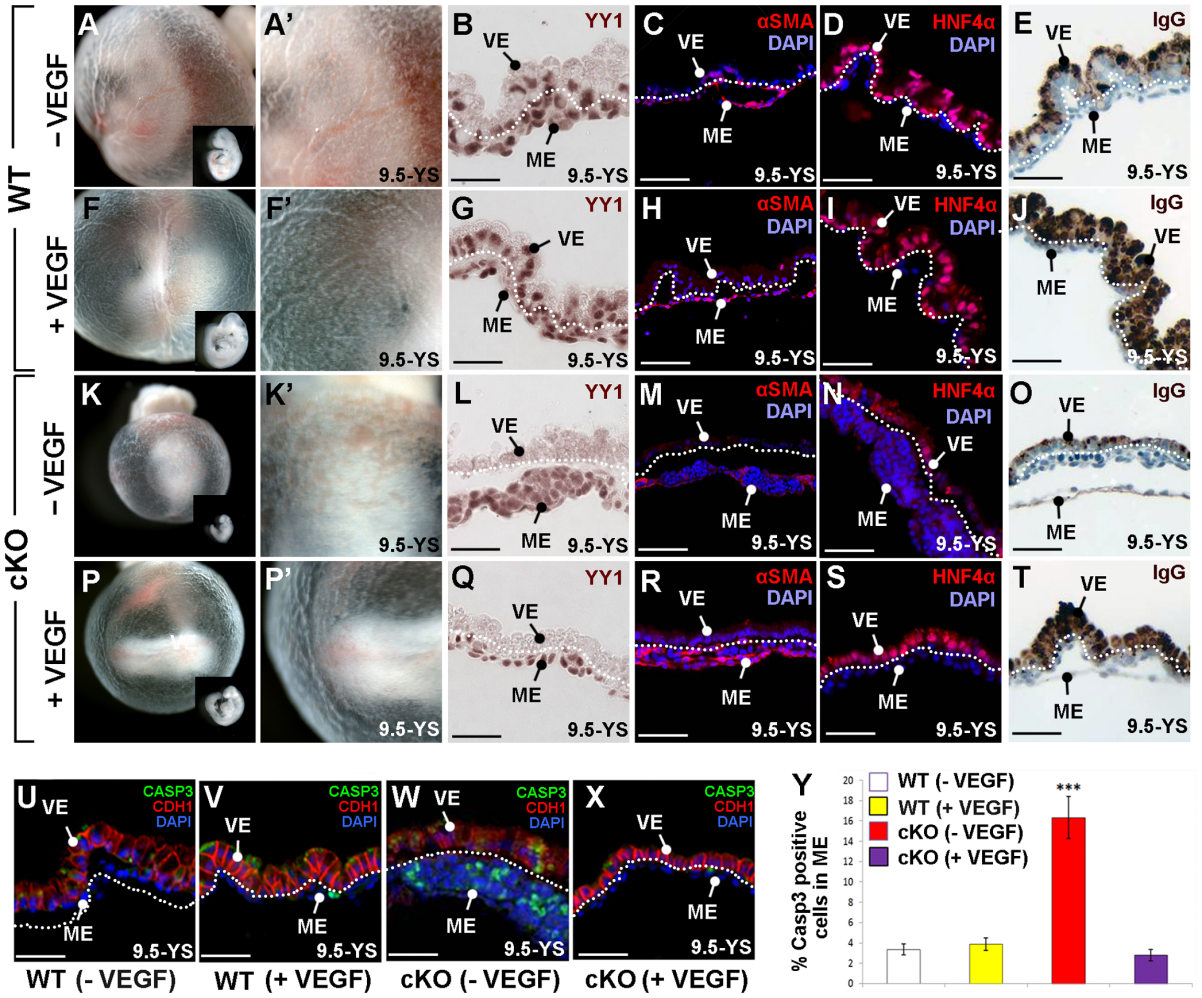
Exogenous VEGF rescues *Yy1* cKO yolk sac defects. A–Y) WT and cKO embryos cultured from 8.5–9.5* dpc* in the presence (+VEGF) or absence (−VEGF) of VEGF. A–E) WT cultured embryos display normal yolk sac vasculature (A–A’), typical embryonic size (inset in A) and the presence of YY1 (brown) in the visceral endoderm (VE) and in yolk sac mesoderm (ME; B). In WT yolk sac sections, αSMA (red) surrounds mature vessels (C), HNF4α (red) is expressed in the visceral endoderm (D) and IgG is localized to the apical visceral endoderm (E). F–J) WT embryos cultured with exogenous VEGF display robust yolk sac vasculature (F, F’), YY1 expression in both YS layers (G), normal αSMA in mature vessels (H), typical HNF4α in the visceral endoderm (I) and high levels of apical IgG (J). K–O) Cultured cKO embryos demonstrate poor vascular development, including pooled blood in the proximal yolk sac (K–K’), no YY1 in the visceral endoderm (L), reduced αSMA (M), reduced HNF4α (N) and decreased apical IgG (O) when compared with WT cultured embryos (A–E). P–T) cKO embryos cultured with exogenous VEGF display normal yolk sac vasculature (P–P’) and increased embryo size (inset in P) when compared to cKO embryos cultured without exogenous VEGF (K–K’). cKO embryos cultured with VEGF lack visceral endoderm YY1 (Q) but have increased αSMA in the yolk sac mesoderm (R) and increased levels of HNF4α (S) and apical IgG (T) in the visceral endoderm when compared to untreated cKO embryos (M–O). U–Y) Immunofluorescence against cleaved Caspase-3 (CASP3, green) and CDH1 (red) of sectioned yolk sacs revealed that typical CDH1 expression found in WT (U) and WT cultured with VEGF (V), was downregulated in cultured cKO embryos but more normal visceral endoderm expression restored when cKO embryos were cultured with VEGF (X). Y) Quantification of cleaved Caspase-3 positive cells demonstrates that the addition of VEGF to cKO embryos restores WT levels of apoptosis. *** = p<0.001; error bars = standard error; dotted line is drawn between the visceral endoderm (VE) and mesoderm derivatives (ME) on yolk sac sections.

Because loss of YY1 mimics many aspects of the VEGFA heterozygous phenotype and because the cKO yolk sacs display reduced VEGFA, we next sought to determine if exogenous VEGF could rescue the cKO yolk sac phenotype. Entire litters containing cKO and WT embryos were dissected at 8.5 *dpc* and cultured *ex vivo* until they were 9.5 *dpc* in the absence (−VEGF, Fig. 6A–E, K–O) or presence of exogenous VEGF (+VEGF, Fig. 6F–J, P–T). Cultured WT embryos exhibit normal growth and yolk sac development (Fig. 6A–E), while addition of VEGF to WT embryos resulted in prominent yolk sac vascularization, an increase in apical vesicle accumulation of IgG and a slightly larger embryo (Fig. 6F–J).

cKO embryos cultured from 8.5 through 9.5 *dpc* are similar to those found *in vivo* (Fig. 6K–O). One difference is that while the *in vivo* developed mutants contained a few prominent dilated vessels (Fig. 1J, 4D), cKO cultured embryos contained many small non-contiguous clusters of dilated vessels. Those in the proximal yolk sac were typically filled with nucleated blood cells (Fig. 6K–N).

Culture of cKO embryos with exogenous VEGF rescued several features of the mutant phenotype. VEGF treated cKO embryos produce a more normal distribution of vascular tissue and larger embryos when compared with cKO embryos cultured in media alone (compare Fig. 6K, K’ to P, P’), despite the absence of YY1 in the visceral endoderm (Fig. 6Q). Furthermore, αSMA expression is restored to normal levels in VEGF treated cKO mesoderm (compare Fig. 6M to R). Surprisingly we find that both HNF4α (Fig. 6S) and IgG levels (Fig. 6T) are restored in the VEGF supplemented cKO visceral endoderm. CDH1, which is reduced in mutant visceral endoderm isolated *in vivo* (Fig. 2P), is similarly reduced in cultured cKO visceral endoderm when compared with cultured WT controls (compare Fig. 6W to U–V). Normal levels of CDH1 are restored when cKO embryos are supplemented with VEGF (compare Fig. 6X to U–V). Finally, we examined apoptosis in the mesoderm of cultured embryos and found that addition of VEGF to the cKO yolk sac results in apoptosis levels that are similar to those of WT embryos (Fig. 6Y).

These results demonstrate that addition of VEGF to cKO embryos rescues both the vascular/mesoderm phenotypes and the visceral endoderm phenotypes. Because the visceral endoderm does not harbor either of the essential early VEGF receptors (*Flt1*, *Flk1*; Fig. S3) and thus cannot directly receive VEGF signals, our results suggest that the visceral endoderm receives a VEGF-responsive signal from the yolk sac mesoderm to maintain visceral endoderm characteristics. Finally, these data support the notion that the main cause of the early yolk sac failure in *Yy1* cKO embryos is the reduction of VEGF.

### Inhibition of VEGF Signaling

**Figure 7:**
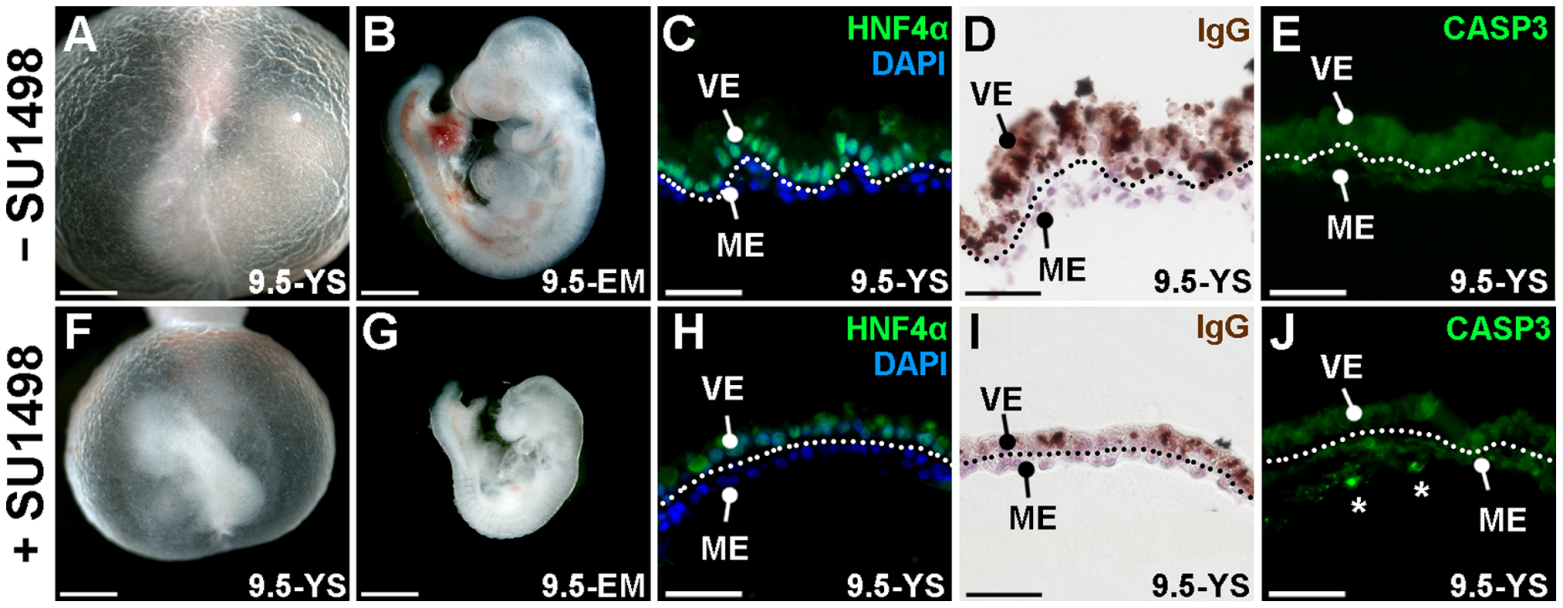
Inhibition of FLK1 in WT embryos results in yolk sac defects similar to *Yy1* cKO. A–J) WT 8.5 *dpc* embryos cultured until they reached 9.5* dpc* in the absence (A–E; −SU1498) or presence of the small molecule SU1498 (F–J; +SU1498). Compared with control embryos (A–E), SU1498 treated embryos displayed clear yolk sac abnormalities, including pooled blood in the proximal yolk sac (F), a small embryo (G), reduced HNF4α (H), reduced apical IgG localization (I) and higher amounts of cleaved Caspase-3 (CASP3) staining (J). Asterisks in J indicate cleaved Caspase-3 positive cells; dotted line represents the division between the yolk sac mesoderm (ME) and visceral endoderm (VE).

To confirm that many of the visceral endoderm defects observed in the cKO yolk sac is due to the loss of VEGF-signaling in the yolk sac mesoderm, we cultured WT embryos with the small-molecule SU1498, which blocks the tyrosine kinase activity of the VEGF receptor, FLK1. Addition of SU1498 to WT 8.5 *dpc* embryos that were then cultured for ∼28 hours resulted in poor yolk sac development, delayed embryo growth and increased apoptosis in the mesoderm derivatives of the yolk sac when compared to embryos cultured in the absence of inhibitor (compare Fig. 7A–B to F–G and E to J). Furthermore we find that SU1498-treated embryos have reduced levels of HNF4α (Fig. 7H) and reduced accumulation of IgG (Fig. 7I) compared to untreated controls (Fig. 7C–D). These results demonstrate that blocking VEGF-receptor mediated signaling in the yolk sac mesoderm not only produces defective blood vessel development but also leads to a loss of visceral endoderm characteristics, supporting the hypothesis that loss of a VEGF-dependant paracrine signal from the yolk sac mesoderm is essential for maintaining the visceral endoderm.

## Discussion

**Figure 8:**
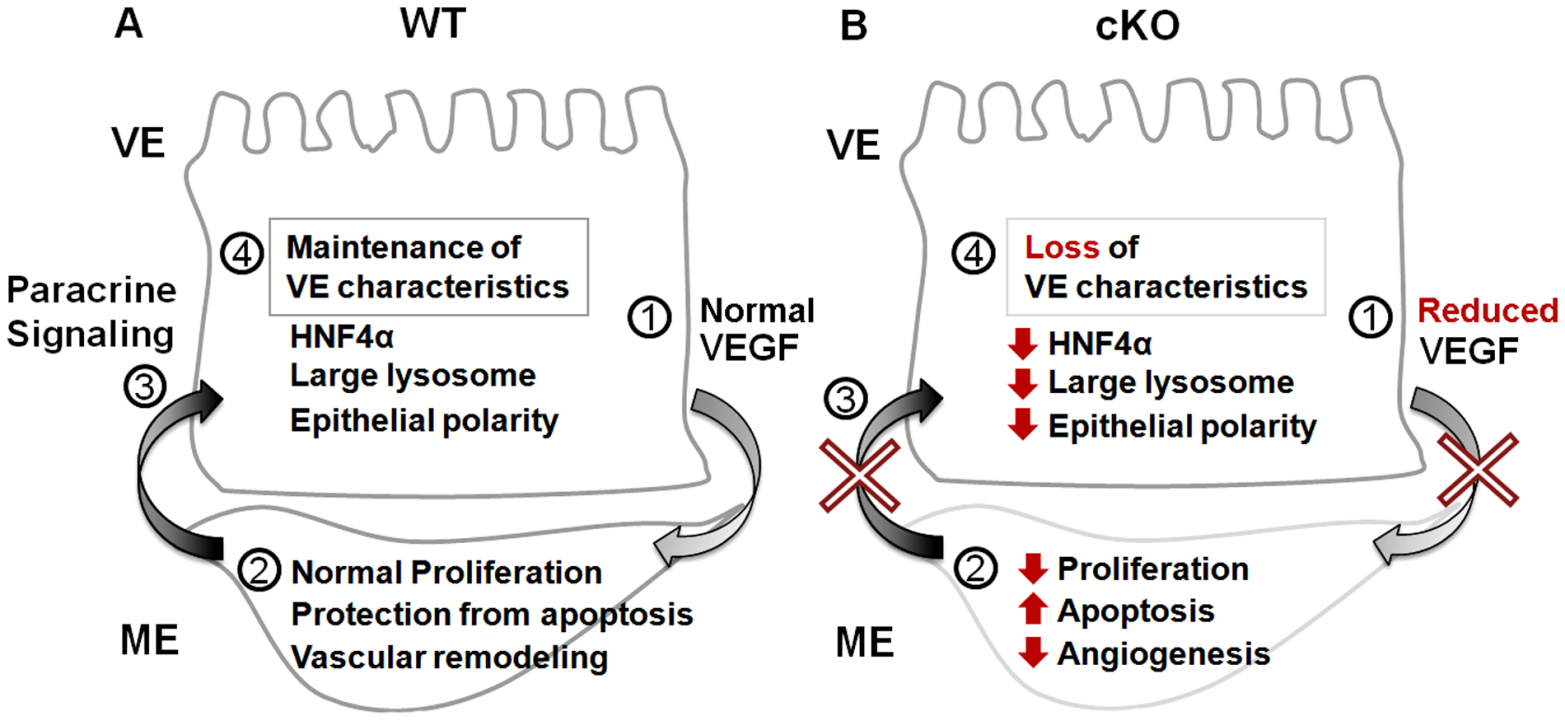
Loss of YY1 leads to defects in paracrine signals necessary for angiogenesis and visceral endoderm integrity. A, B) A summary of the signaling events downstream of YY1 in WT and mutant yolk sacs. A) In the presence of YY1, normal VEGF levels produced by the visceral endoderm (VE) allow the underlying mesoderm derivatives (ME) to undergo events associated with vascular remodeling. The underlying vascular tissue is the source of a VEGF-dependant paracrine signal(s) that is required by the visceral endoderm to maintain characteristics such as epithelial polarity, large apical lysosomes and HNF4α expression. B) In the absence of YY1 in the visceral endoderm, decreased levels of paracrine VEGF result in defective angiogenesis, increased apoptosis and decreased proliferation in the adjacent mesoderm. Because of reduced VEGF signaling, the yolk sac mesoderm does not generate the paracrine signal(s) needed to maintain epithelial characteristics in the visceral endoderm, resulting in decreased HNF4α, a loss of large lysosomes and reduced CDH1 levels.

Here we assessed the developmental role of *Yy1* in the extraembryonic and embryonic endoderm. While YY1 is not required for embryonic endoderm-derived organ specification, we find that YY1 is essential in the visceral endoderm to support angiogenesis in the adjacent mesoderm. Between 9.0–9.25 *dpc* mutants display a loss of VEGFA and a reduction of cell polarity markers, a lack of large apical lysosomes and the reduced expression of several visceral endoderm-expressed genes, including *Pgc1α*, *vHNF1* and *Hnf4α.* These defects are accompanied by decreased proliferation and increased apoptosis in the adjacent yolk sac mesoderm. Exogenous VEGF rescued both the angiogenesis defects and many of the visceral endoderm phenotypes including restoration of epithelial polarity, large apical lysosomes and HNF4α expression. Inhibition of VEGF receptor signaling in the yolk sac mesoderm produces phenotypes not only in the mesoderm but also in the visceral endoderm. Taken together these data suggest: 1) that one role of YY1 in the early visceral endoderm is to regulate VEGFA translation or protein stability and 2) that a VEGFA-responsive paracrine signal generated by the yolk sac mesoderm is important for the maintenance of visceral endoderm characteristics (Fig. 8).

### A VEGF-responsive Paracrine Signal from the Mesoderm is Necessary for Visceral Endoderm Function

It is clear that VEGF produced in the visceral endoderm is required for angiogenesis in the yolk sac mesoderm [18]. We were thus not surprised that exogenous VEGF restored the angiogenesis defects observed in our mutant yolk sacs. We were intrigued to find that many of the visceral endoderm phenotypes observed in our cKO embryos are also restored by the addition of exogenous VEGF, suggesting that the visceral endoderm phenotype is not directly due to the loss of YY1 in this tissues. While it is possible that the visceral endoderm defects are caused by a cell autonomous role of VEGF in the visceral endoderm, the exclusive expression of the critical early embryonic VEGF receptors, *flk1* and *flt1*, specifically in the yolk sac mesoderm [20], [22], [23] argues against this possibility. Instead we suggest that the defects observed in the visceral endoderm are due to a loss of a paracrine signal produced by the VEGF-responsive yolk sac mesoderm. For example, *Hnf4α* plays an essential role in the visceral endoderm for normal yolk sac development [38], [39], [42], [43], [44], [45]. Exogenous VEGF restores HNF4α in cKO embryos, suggesting that YY1 is not required for its expression. Furthermore we demonstrate that inhibition of the FLK1 in WT embryos causes visceral endoderm phenotypes similar to YY1 cKO (Fig. 7). Finally, it is interesting to note that exogenous VEGF causes an apparent increase in apical IgG localization and CDH1 even in WT embryos (compare Fig. 6E to J, and U to V), supporting the hypothesis that VEGF signals received by the yolk sac mesoderm influences visceral endoderm phenotypes independent of YY1.

A survey of the mutations that reduce VEGF expression in the yolk sac and result in yolk sac angiogenesis defects, including *VegfA* heterozygotes, *VegfA* hypomorphic and *Arnt* homozygous null embryos, reveals visceral endoderm phenotypes similar to that of *Yy1* cKO [15], [18], [46]. Similarly a survey of the mutations that alter the ability of the yolk sac mesoderm to respond to VEGF signaling produce a similar visceral endoderm phenotypes [22], [47]. Taken together, these observations further support our hypothesis that a VEGF-responsive paracrine signal is essential to maintain visceral endoderm integrity.

### YY1 Modulation of VEGF

Recent studies have found that YY1 directly binds and activates human *VegfA*, *B* & *C* promoters [48]. Contrary to this data we find that *VegfA* mRNA is not depleted in the cKO yolk sac, but we do see a gradual reduction in VEGFA protein in the visceral endoderm between 9.0–9.5 *dpc*. Taken together with the VEGF rescue experiments, our data suggest that a reduction in VEGF protein levels is the cause of the cKO yolk sac phenotypes.

One intriguing question is how does YY1 modulate VEGF levels if not at the transcriptional level? Loss of YY1 in skeletal muscle results in a significant reduction in *Pgc1α* expression [40]. Similarly, in our cKO visceral endoderm we find a dramatic reduction of *Pgc1α* expression. *Pgc1α*-deficient mice develop normally, exhibiting a phenotype upon physiological stress, suggesting that loss of *Pgc1α* is not the cause of early embryonic lethality in *Yy1* cKO embryos [49], [50]. A recent report has shown that with activity-induced stress, *Pgc1α-*deficient skeletal muscle exhibits a loss of VEGFA protein with no loss of *VegfA* transcripts [51], suggesting that *Pgc1α* loss could play a role in the VEGFA reduction observed in our cKO yolk sacs.

### Role of YY1 in Paracrine Signaling *in vivo*

Recent *in vivo* studies have shown an essential role for *Yy1* in the regulation of secreted growth factors during development, including *Bmp15* and *Gdf9* during normal oocyte development [7] and *Lefty-2* during gastrulation [8]. In these tissues a loss of YY1 results in defective paracrine signaling of key secreted growth factors, producing defects in adjacent tissues that normally receive the signal. The work presented here is another example demonstrating that YY1 acts as a critical regulator of paracrine signals during development and highlights the importance of *in vivo* functional studies.

## Supporting Information

Figure S1 *FoxA3-Cre* activity monitored with the *R26R* allele. A–F) *R26R*;*FoxA3-Cre* double heterozygotes were dissected at the stages indicated and *Cre* activity monitored by *LacZ* staining (blue). Whole mount images (A–C) and eosin counterstained transverse sections (D–F) of the same embryo at the indicated plane (green line, A–C). A, D) At 7.5 *dpc*, *LacZ* expression is confined to and mosaic within in the visceral endoderm (VE) and not yet found in the early definitive endoderm (bottom white portion in A). B,E) At 8.5 *dpc LacZ* activity is found throughout the visceral endoderm of the yolk sac and is mosaic within the definitive endoderm (DE). C, F) *LacZ* expression is found throughout the definitive endoderm including the liver bud (LB) at 9.5* dpc*. ME = yolk sac mesoderm.
(TIF)

Figure S2 Pancreas specification in *Yy1* cKO definitive endoderm. A–D) Immunofluorescence of transverse sections of WT (A–B) and cKO (C–D) 9.5 *dpc* embryos using YY1 (red), PDX1 (green) and the nuclear stain DAPI (blue). Co-expression of all 3 markers (yellow) is found in both the ventral (A) and dorsal pancreas buds (B) in WT embryos. C–D) Despite the loss of YY1 in the definitive endoderm (blue cells adjacent to asterisk) and its derivatives, both the ventral pancreas (green, C) and dorsal pancreas (green, D) express PDX1. VP = ventral pancreas bud; DP = dorsal pancreas bud.
(TIF)

Figure S3 Yolk sac separation reveals layer-specific gene expression patterns. cDNA obtained from WT 9.5 *dpc* yolk sacs isolated whole (YS) or separated into visceral endoderm (VE) and mesoderm (ME). RT-PCR reveals that *VegfA* is expressed mainly in the VE. *Hnf4α* and *Pgc1α* are expressed exclusively in the visceral endoderm while the VEGF receptors, *Flt1* and *Flk1*, are confined to the mesoderm layer. *β-actin* expression was used as a loading control.
(TIF)

Table S1 A complete list of all traditional RT-PCR primers used.
(DOC)
