# Supplementary figures and images for: Visceral Endoderm Expression of Yin-Yang1 (YY1) Is Required for VEGFA Maintenance and Yolk Sac Development
Source: PLoS One. 2013 Mar 15;8(3):e58828. doi: 10.1371/journal.pone.0058828 (PMC3598950; doi:10.1371/journal.pone.0058828)

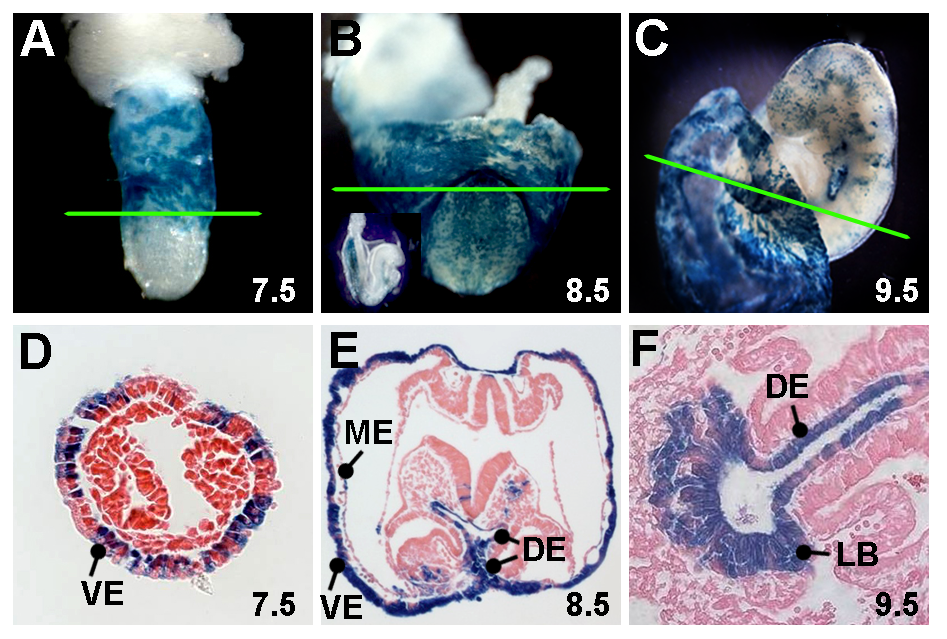

Supplement: Figure S1 — FoxA3-Cre activity monitored with the R26R allele. A–F) R26R;FoxA3-Cre double heterozygotes were dissected at the stages indicated and Cre activity monitored by LacZ staining (blue). Whole mount images (A–C) and eosin counterstained transverse sections (D–F) of the same embryo at the indicated plane (green line, A–C). A, D) At 7.5 dpc, LacZ expression is confined to and mosaic within in the visceral endoderm (VE) and not yet found in the early definitive endoderm (bottom white portion in A). B,E) At 8.5 dpc LacZ activity is found throughout the visceral endoderm of the yolk sac and is mosaic within the definitive endoderm (DE). C, F) LacZ expression is found throughout the definitive endoderm including the liver bud (LB) at 9.5 dpc. ME = yolk sac mesoderm. (TIF) [file pone.0058828.s001.tif]

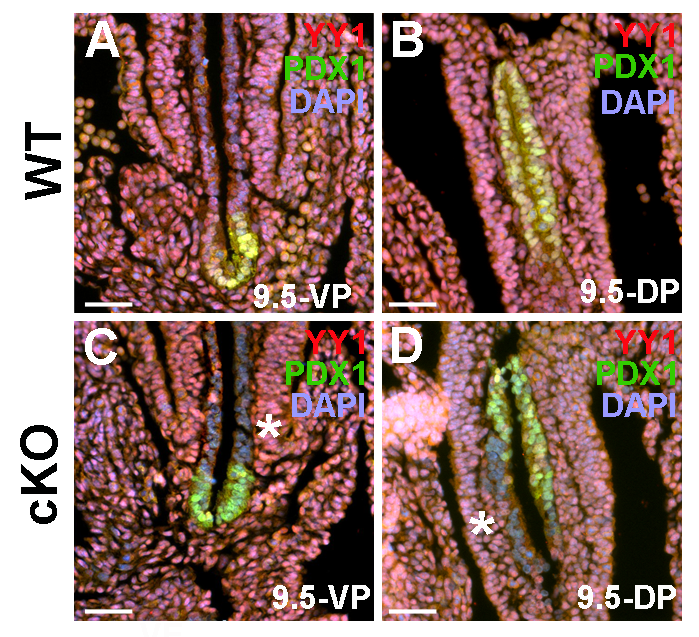

Supplement: Figure S2 — Pancreas specification in Yy1 cKO definitive endoderm. A–D) Immunofluorescence of transverse sections of WT (A–B) and cKO (C–D) 9.5 dpc embryos using YY1 (red), PDX1 (green) and the nuclear stain DAPI (blue). Co-expression of all 3 markers (yellow) is found in both the ventral (A) and dorsal pancreas buds (B) in WT embryos. C–D) Despite the loss of YY1 in the definitive endoderm (blue cells adjacent to asterisk) and its derivatives, both the ventral pancreas (green, C) and dorsal pancreas (green, D) express PDX1. VP = ventral pancreas bud; DP = dorsal pancreas bud. (TIF) [file pone.0058828.s002.tif]

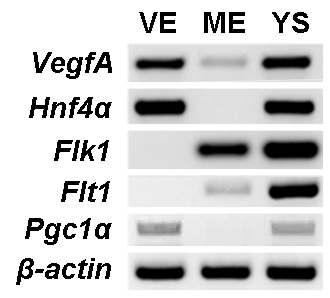

Supplement: Figure S3 — Yolk sac separation reveals layer-specific gene expression patterns. cDNA obtained from WT 9.5 dpc yolk sacs isolated whole (YS) or separated into visceral endoderm (VE) and mesoderm (ME). RT-PCR reveals that VegfA is expressed mainly in the VE. Hnf4α and Pgc1α are expressed exclusively in the visceral endoderm while the VEGF receptors, Flt1 and Flk1, are confined to the mesoderm layer. β-actin expression was used as a loading control. (TIF) [file pone.0058828.s003.tif]
