# Supplementary material for: Visceral Endoderm Expression of Yin-Yang1 (YY1) Is Required for VEGFA Maintenance and Yolk Sac Development
Source: PLoS One. 2013 Mar 15;8(3):e58828. doi: 10.1371/journal.pone.0058828 (PMC3598950; doi:10.1371/journal.pone.0058828)
Supplement: Table S1 — A complete list of all traditional RT-PCR primers used. (DOC) [file pone.0058828.s004.doc]

Supporting Infomation Table 1. A complete list of all traditional RT-PCR primers used.

| Gene | Forward primer (5’-3’) | Reverse primer (5’-3’) |
| --- | --- | --- |
| *Hprt* | TTGGGCTTACCTCACTGCTTTC | CCTGGTTCATCATCGCTAATCAC |
| *β-actin* | ACGGCCAGGTCATCACTATTG | ATGGATGCCACAGGATTCCAT |
| *Yy1* | ACGACGACTACATAGAGCAGACG | ACGAACGCTTTGCCACACT |
| *VegfA (ex3-8)* | GTAACGATGAAGCCCTGGAGTG | TGAGAGGTCTGGTTCCCGAAA |
| *VegfA (ex2-3)* | TTACGATGAAGCCCTGGAGTG | TGACCCCCTAAATGCTACCTAAGAG |
| *Hif1α* | TGCTCATCAGTTGCCACTTCCC | CCATCTGTGCCTTCATCTCATCTTCTT |
| *Hnf4α* | AGGTTTAGCCGACAATGTGTG | TCATCCAGAAGGAGTTCGCAGT |
| *vHnf1* | GACACTCCTCCCATCCTCAAAG | GCCCTGTTACACTCCTCCACTA |
| *Ihh* | TGGGTGTATTACGAGTCCAAGGC | TGCTGGTTCTGTATGATTGTCCG |
| *Ttr* | AGAGTAGAACTGGACACCAAATCG | GGAGCAGGGGAGAAAAATGAG |
| *Flk1* | CCAAGGCGACTATGTTTGCTCTG | CCAATGGTTGTTGTCTGATTCTCC |
| *Flt1* | AGATAGGACTGCTGAACTGCGAAG | GTTGAAAGACTGGAACGAGGACC |
| *Gata4* | CAGAAAACGGAAGCCCAAGAAC | TGCCCATAGTGAGATGACAGCC |
| *Gata6* | GCGTAGAAATGCTGAGGGTGAG | GCCGTCTTGACCTGAATACTTGAG |
| *qK* | CAACTTGAAGCAGAAACGGGA | GGTGCCAATGTGTAGGGGTAT |
| *Pgc1α* | GTGTGTCAGAGTGGATTGGAGTTG | ATGTTCGCAGGCTCATTGTTG |
| *Enpp2* | GGAAAATGCCTGTCACTGCTC | GATAGTTAGGATTCTCCGCTCGT |
| *Snx2* | GTGAAGCCCACAGACTTTGAGG | GGTTTGGAGCCATTAGAGTTTGC |
| *Lamp2* | CAACTCCAACTCCAACTCCAACC | GGCACCTTCTCCTCAGTGATGT |
| *Rab7* | GTAACCAGTACAAAGCCACAATAGG | CAACAAAAGGGAAGTTCTCGG |
| *Tfeb* | GGAGCGGCAGAAGAAAGACA | TGGACAGGTTGGGGAATGG |
| *Lrp2* | TCCTTCGTGCCTATGTCTGTG | ATTGGAGCAAGTGAACTGGTGG |
